# Supplementary material for: Investigation of the role of VHL-HIF signaling in DNA repair and apoptosis in zebrafish
Source: Oncotarget. 2020 Mar 31;11(13):1109–30. doi: 10.18632/oncotarget.27521 (PMC7138166; doi:10.18632/oncotarget.27521)
Supplement: Supplementary file 1 [file oncotarget-11-1109-s001.pdf]

Investigation of the role of VHL-HIF signaling in DNA repair and apoptosis in zebrafish

SUPPLEMENTARY MATERIALS

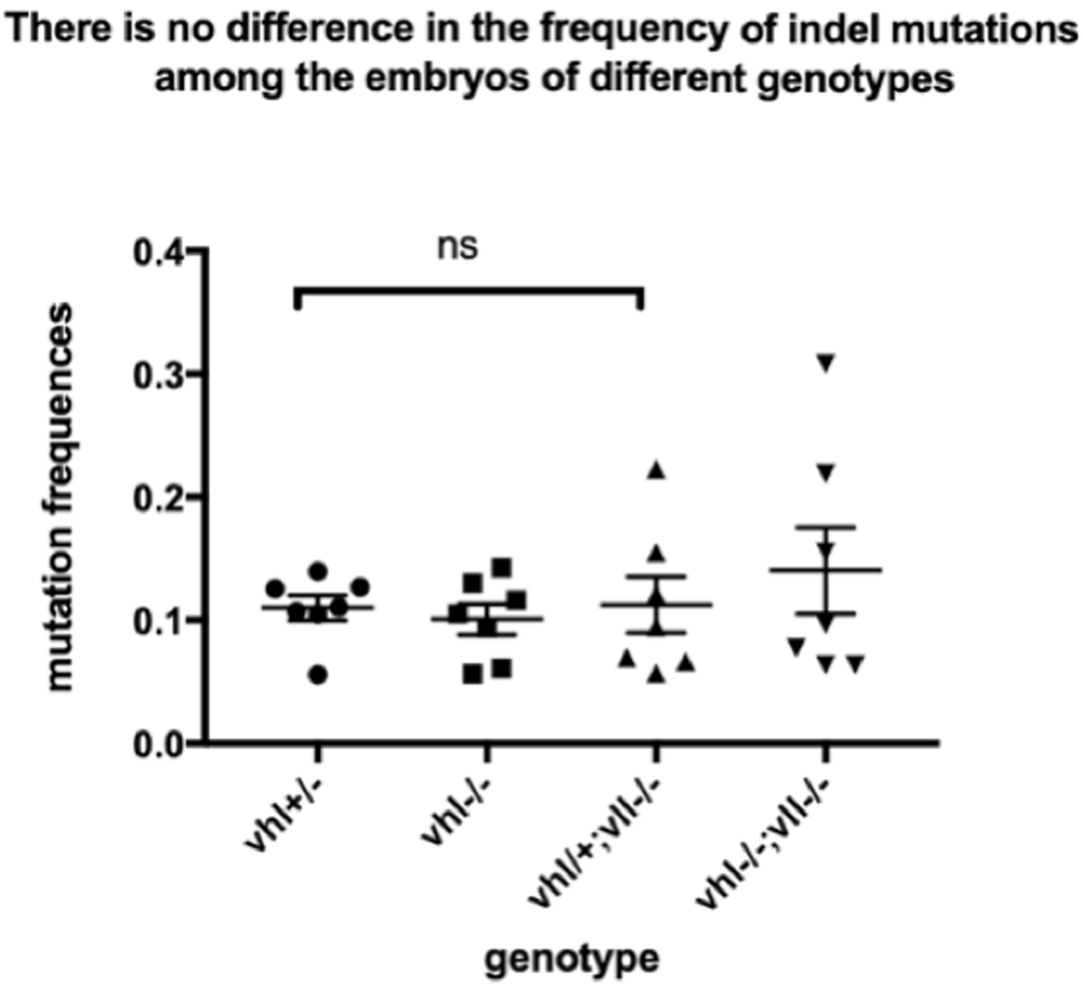

Supplementary Figure 1: There was no difference in the frequency of small indel mutations, created by CRISPR injection against *AR*, among *vhl–/–*, *vhl–/–;vll–/–* embryos and their siblings. Embryos were collected from *vhl+/–* and *vhl+/–;vll–/–* pair mating and were injected with CRISPR targeting *vhl* and *AR*. The genomic DNA around the target sites was amplified by PCR and sequenced by deep sequencing. This revealed that there was no significant difference in small indel mutation frequency between embryos with different genotypes. $^{ns}p > 0.05$, one way ANOVA.

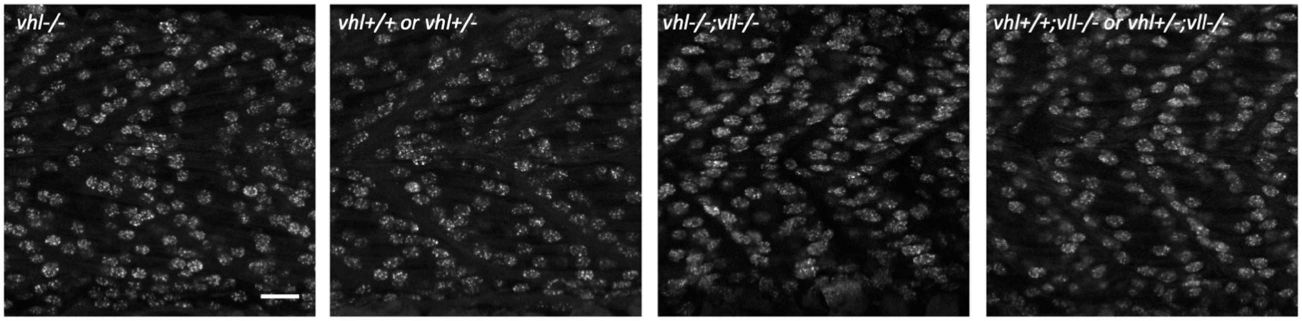

Supplementary Figure 2: γH2AX foci were equally formed in *vhl–/–*, *vhl–/–;vll–/–* embryos and their siblings immediately after the irradiation. The embryos from *vhl+/–* and *vhl+/–;vll–/–* incrosses were collected and irradiated with X-ray at 24hpf. Immediately after the irradiation the embryos were fixed and the γH2AX foci formation was examined. It revealed that all embryos of different genotypes showed equally distributed γH2AX foci formation, indicating the DNA damage is equally introduced in these embryos. Scale bar: 20 μm.

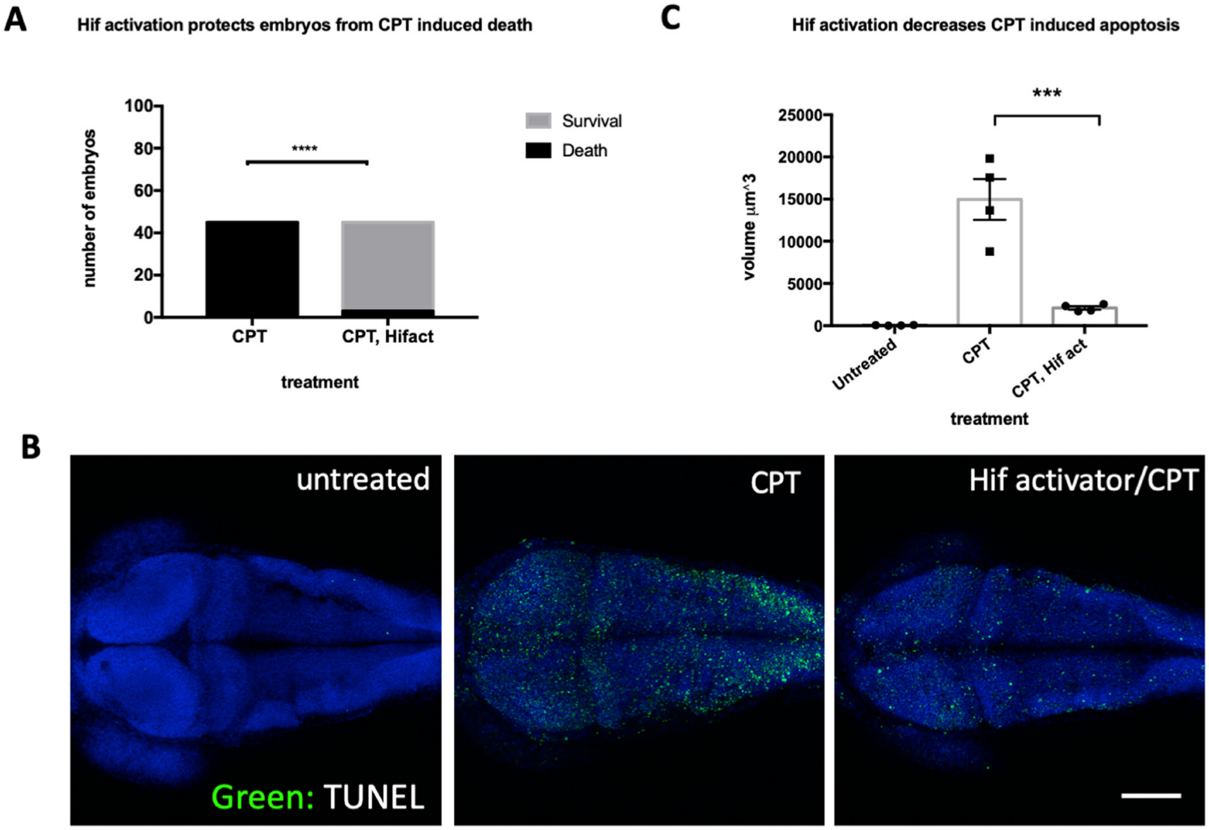

Supplementary Figure 3: Hif activator treated embryos were protected from CPT induced death. (**A**) When the wild type embryos were treated with 20 nM CPT overnight, all embryos died by 5dpf. In contrast, the majority of embryos survived on 5dpf when Hif activator was treated with CPT (see Figure 5E and 5F). $^{****}p < 0.0001$, chi-square test. (**B**) We performed the TUNEL assay with wild type embryos treated with 10 nM CPT with or without the pre-treatment with Hif activator. The embryos were treated with CPT at 32hpf and were fixed at 48hpf. This showed that there was a significant reduction in TUNEL staining in Hif activator treated embryos. The data are quantified in (**C**) $^{***}p < 0.001$, one way ANOVA; Holm-Sidak's multiple comparison test. Scale bar: 0.2 mm.

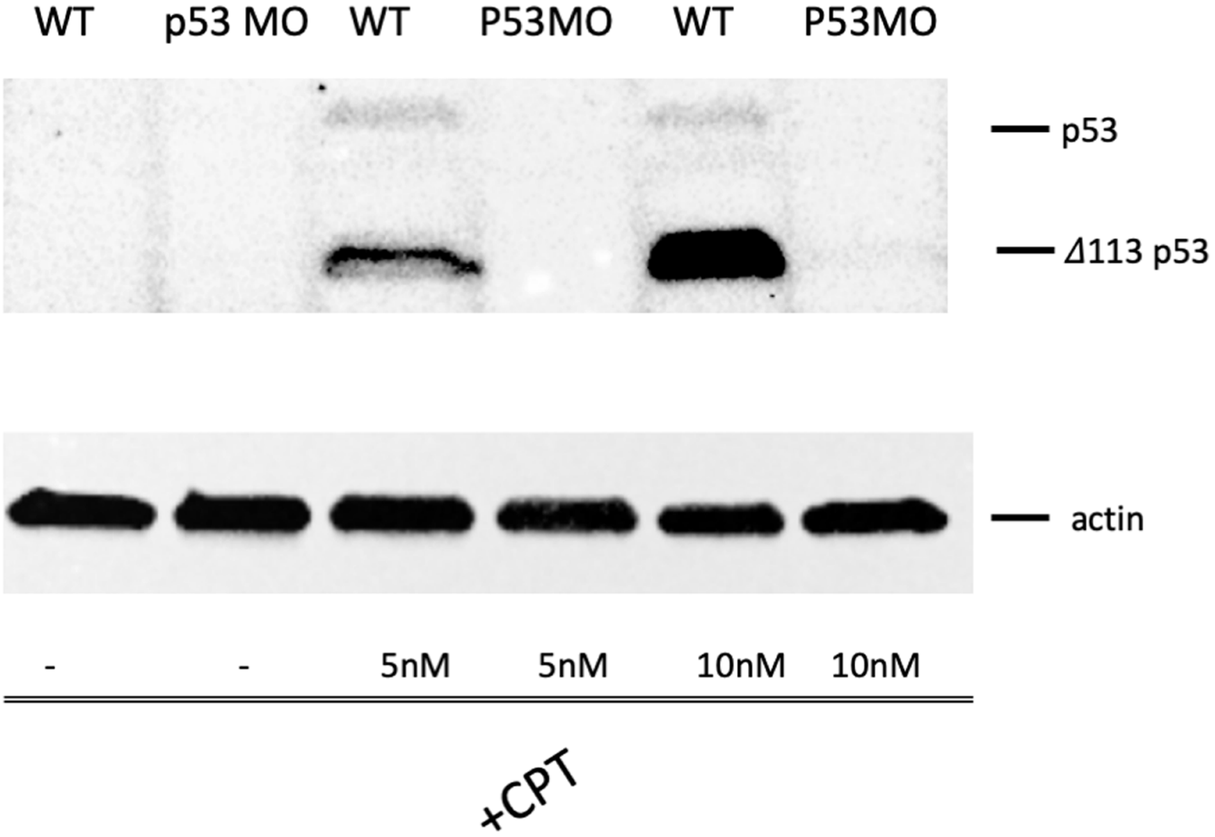

Supplementary Figure 4: Both isoforms of p53, full length and Δ113p53, were efficiently knocked down by p53 morpholino injection in zebrafish embryos. The p53 morpholino injected embryos and uninjected wild type embryos were treated with CPT and the level of p53 induced in the embryos was examined by Western blot. This showed that there was a high level of p53 induction in the CPT treated wild type embryos. In contrast, the induction of both isoforms of p53 was dramatically suppressed in the p53 morpholino injected embryos, demonstrating the efficiency of p53 morpholino.

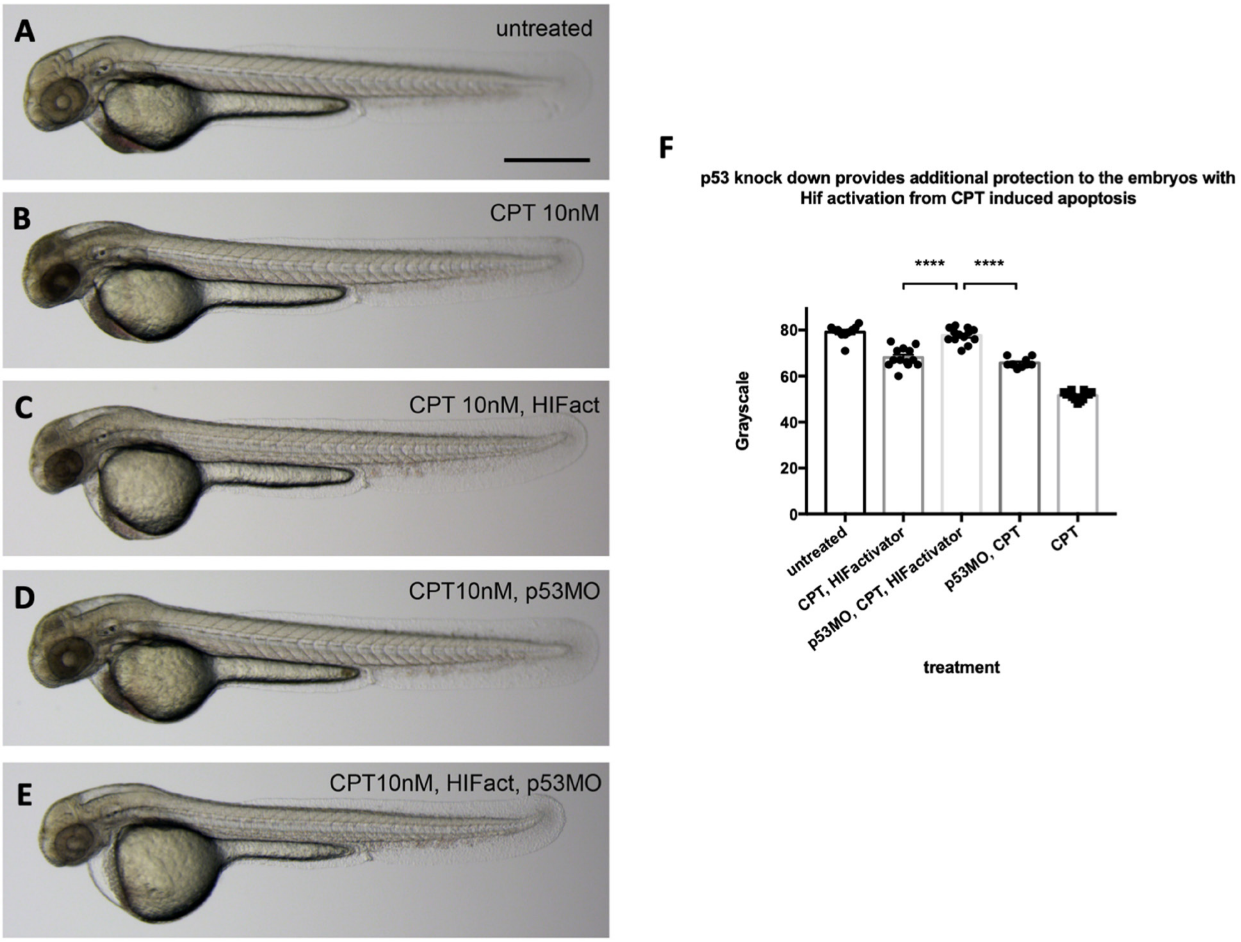

Supplementary Figure 5: p53 knock down enhanced the protective effect of Hif activation in response to CPT treatment. To test whether p53 knock down enhances the protective effect of Hif activation in response to CPT treatment, we injected p53 morpholino before the Hif activator and CPT treatment. (**A**) untreated control, (**B**) CPT treated control. We found that p53 knock down led to a further protection for Hif activated embryos from CPT induced apoptosis (compare **D** and **E**). We also found that Hif activation enhanced the protective effect of p53 knock down in response to CPT treatment (compare **C** and E). The data are quantified in (**F**) $^{****}p < 0.0001$, one way ANOVA; Holm-Sidak's multiple comparison test. Scale bar: 0.5 mm.

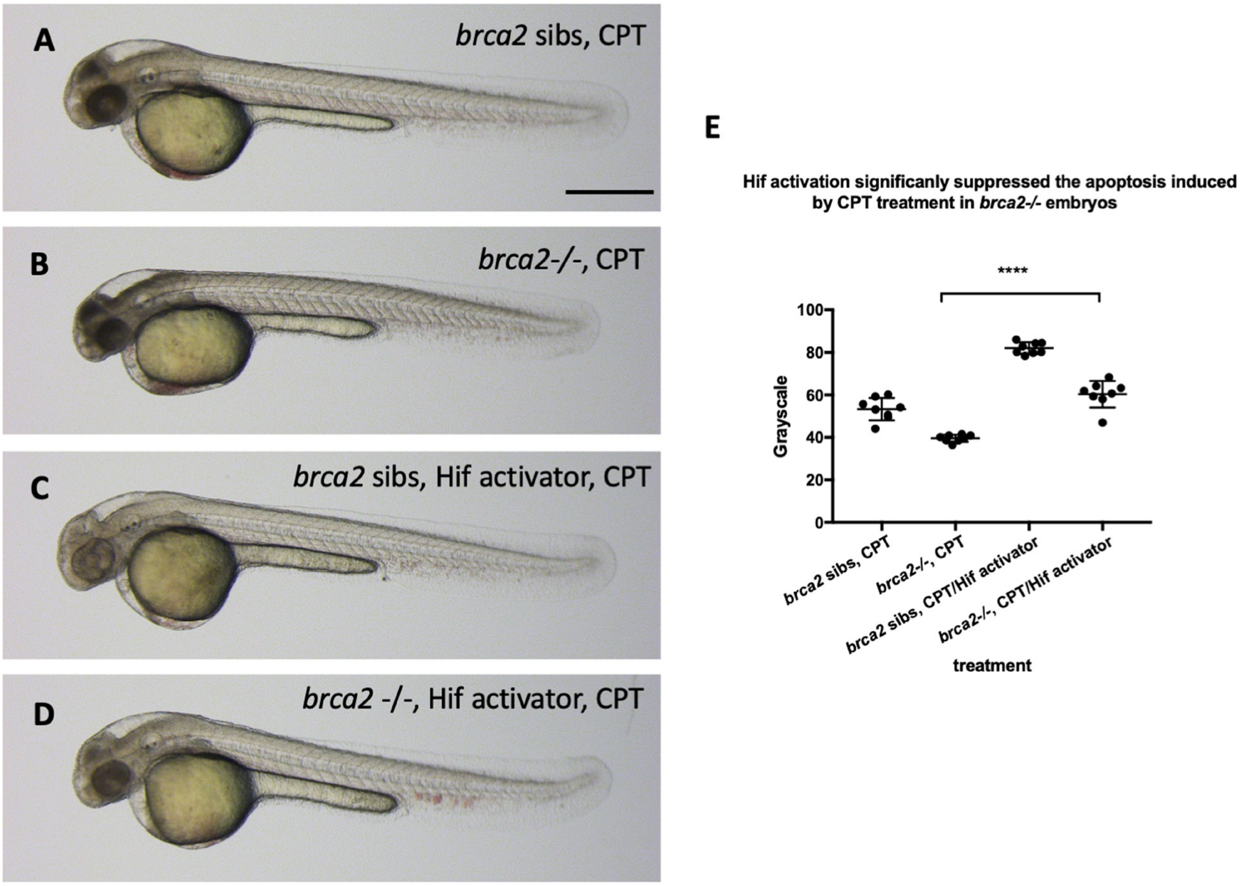

Supplementary Figure 6: Hif activation protects *brac2–/–* embryos from CPT induced apoptosis. To confirm that the protection of *brca2–/–;vhl–/–;vll–/–* embryos from the X-ray treatment was due to the activated Hif in the embryos, we activated Hif by chemical treatment in *brca2–/–* embryos and subjected them to CPT treatment. (**A**) Sibling embryo CPT-treated, (**B**) *brca2–/–* CPT-treated, (**C**) Sibling embryo CPT-treated, with Hif activator, (**D**) *brca2–/–* CPT-treated, with Hif activator. This revealed that Hif activator treatment significantly suppressed the apoptosis induced by CPT treatment in the *brca2–/–* embryos (compare B and D). The data are quantified in (**E**) $^{****}p < 0.001$, one way ANOVA; Holm-Sidak's multiple comparison test. Scale bar: 0.5 mm.

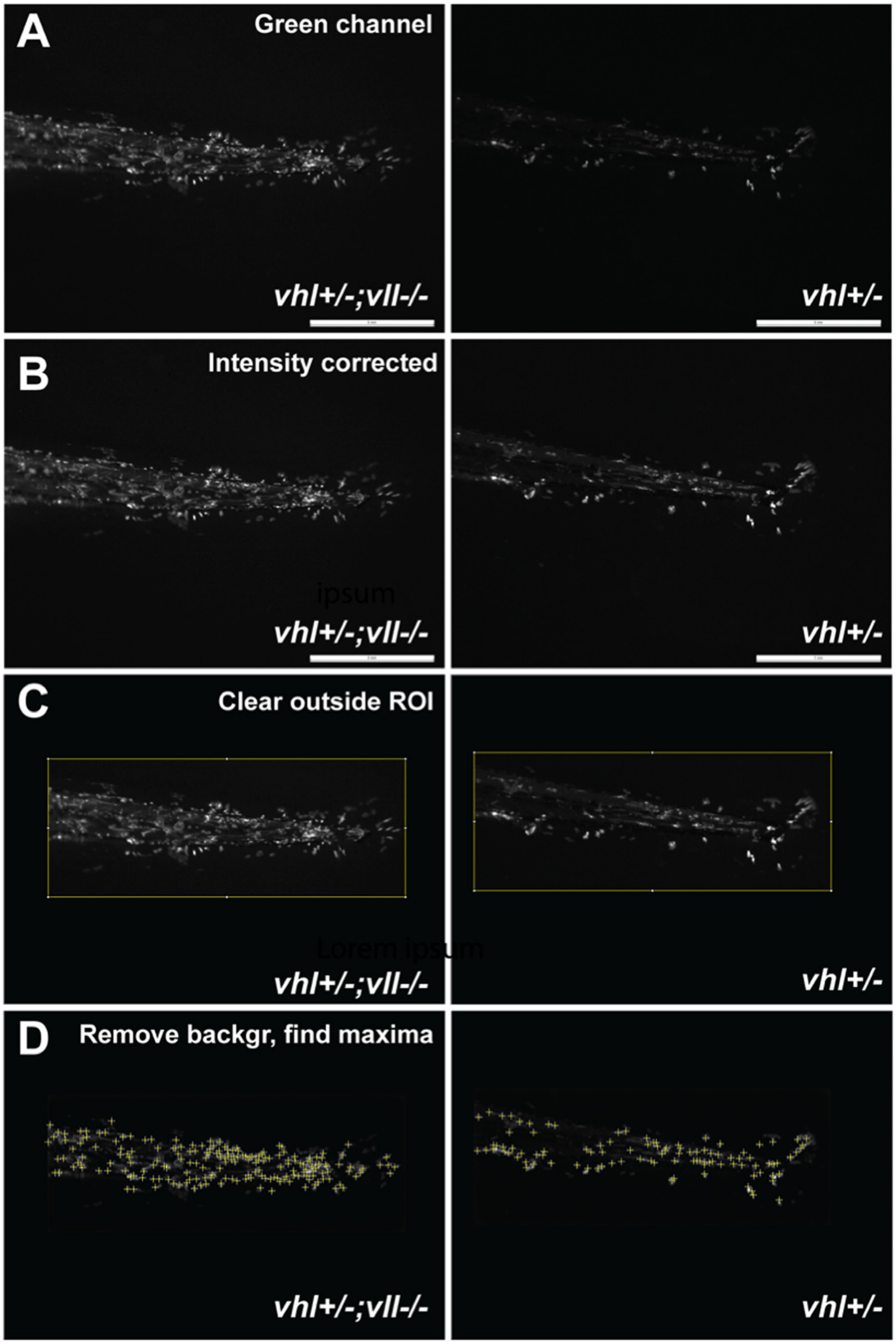

Supplementary Figure 7: The intensity of EGFP levels was normalised for the LOH analysis. In order to make a comparison of the levels of LOH, we first corrected for a lower level of EGFP that is observed in *vhl–/– vs vhl–/–;vll–/–* LOH cells. This was done in FIJI/ImageJ by splitting the colour images in individual channels for measuring the EGFP intensity in the green channel (**A**). We then measured well-defined isolated fin fibroblast LOH cells in 14 different images of each genotype, and determining the grey value of these cells using the "*wand tool*" with a tolerance of 50 to select the cell. This gave values of 101 and 57 as average brightness for *vhl–/–;vll–/–* and *vhl–/–* LOH cells, respectively. In order to equalise the intensity of the images, we therefore multiplied the resulting *vhl–/–* grayscale images by 1.78 to match the intensities of *vhl–/–;vll–/–* using "*math; multiply*" function (**B**). For an estimation of the number of LOH cells, a standard box (coordinates 0450-0696) is drawn around the tail and saved to the ROI manager, the image is cleared outside using "*edit; clear outside*" (**C**). Unequal background is removed with *process; subtract background* (rolling ball radius: 50 pixels), then the *"process; find maxima"* function was used to estimate the number of LOH cells. Proper sensitivity was set using a sample image, and kept constant afterwards for all images (Prominence > 40 for **D**).

Supplementary Table 1: A qPCR with *rps29* as a reference gene also suggests the synergistic effect of the loss of Vhl and Vll in the Hif regulation

Gene Name	Gene Symbol	*vll* vs *wt*	*vhl* vs *wt*	*vhl;vll* vs *wt*
egl-9 family hypoxia-inducible factor 3	*egln3*	0.76	79.43	123.43
vascular endothelial growth factor Aa	*vegfaa*	0.82	2.75	2.78
aminolevulinate, delta-, synthase 2	*alas2*	1.58	8.87	12.32
MAP kinase interacting serine/threonine kinase 2b	*mknk2b*	0.82	2.01	2.83
GATA binding protein1a	*gata1a*	1.37	3.61	2.79

We repeated qPCR to examine the expression level of a few Hif target genes with *rps29* as a reference gene instead of previous *β-actin*. In contrast to the previous qPCR results, the changes in Hif target gene expression was not observed in *vll* mutants. However, a further upregulation of Hif target genes was consistently observed in *vhl–/–;vll–/–* mutants. The values are fold changes, and they are means of three technical and three biological replicates.
